# Usefulness of Hamilton rating scale for depression subset scales and full versions for electroconvulsive therapy

**DOI:** 10.1371/journal.pone.0259861

**Published:** 2021-11-09

**Authors:** Caoimhe Fenton, Declan M. McLoughlin

**Affiliations:** 1 Department of Psychiatry, Trinity College Dublin, St Patrick’s University Hospital, Dublin, Ireland; 2 Trinity College Institute of Neuroscience, Trinity College Dublin, Dublin, Ireland; Yale School of Medicine, UNITED STATES

## Abstract

**Objectives:**

We investigated the predictive value of subset scales and full versions of the Hamilton Rating Scale for Depression (HAMD) for therapeutic outcomes in ECT.

**Methods:**

This secondary analysis of patients with major depression (N = 136; 63% female; age = 56.7 [SD = 14.8]) from the EFFECT-Dep trial (NCT01907217) examined the predictive value of Evans-6, Toronto-7, Gibbons-8 and Maier-Philip 6 HAMD subset scales and three ‘full’ versions (HAMD-17, HAMD-21 and HAMD-24) on therapeutic outcomes. We also examined early improvement on subset scales and full versions as predictors of response and remission and explored predictive abilities of individual HAMD-24 items.

**Results:**

The subset scales and full scales lacked sufficient predictive ability for response and remission. Receiver operating characteristic curves identified a lack of discriminative capacity of HAMD subset scales and full versions at baseline to predict response and remission. Only the Maier-Philip-6 was significantly associated with percentage reduction in HAMD-24 scores from baseline to end of ECT course. Early improvement on most of the subset scales and full versions was a sensitive and specific predictor of response and remission. Four of the HAMD-24 items were significantly associated with response and one with remission.

**Conclusions:**

Limited utility of the HAMD subset scales and full versions in this context highlight a need for more tailored depression rating scales for ECT.

## Introduction

Electroconvulsive therapy (ECT) is the most acutely effective intervention for severe, treatment-resistant and sometimes life-threatening depression [1, 2]. Depression severity at baseline predicts response to ECT [3]. In clinical trials of anti-depressant treatments, the Hamilton Rating Scale for Depression (HAMD) has been considered the ‘gold standard’ for assessing depression severity [4]. However, the complete HAMD is time consuming and has been criticised for conceptual and psychometric shortcomings, in particular its multidimensionality and debated sensitivity to mood change [5–7]. Use of total HAMD scores for assessing depression severity, as is common practice in clinical trials, may thus present a methodological mistake. Because of its multidimensionality, the HAMD is relatively insensitive to improvements in classic depressive symptoms such as depressed mood, guilt, and suicidal ideation [8].

To better meet the need for an assessment device capable of measuring depression severity and to resolve the problem of assessment sensitivity, use of unidimensional HAMD subset scales has been proposed [6]. A number of these have been deduced through factor analytic techniques such as the Evans-6 [9], Toronto-7 [10], Gibbons-8 [11] and the Maier-Philip 6-item subset scales [12] (see Table 1). A meta-analysis of eight fluoxetine studies with 1,658 patients showed that the Maier-Philip-6 and the Bech-6 subset scales were more sensitive to change than the full 17-item HAMD [13]. Use of the shorter HAMD subset scales also substantially reduces the time required for rating, about 15–20 min for the 17-item HAMD (HAMD-17) scale compared to 5 minutes for subset scales with 6–8 items [14]. Due to their good ability to detect changes in depressive symptoms and by focusing on core depressive symptoms [5, 6, 15], using briefer HAMD subset scales might be an economic and more precise measure of therapeutic outcomes in clinical trials and practice.✓

**Table 1:** Hamilton depression rating scale (HAMD) subscales and their items.

	Items	Evans-6	MP-6	Toronto-7	Gibbons-8
HAMD item no.					
*1*	*Depressed mood*	✓	✓	✓	✓
*2*	*Feelings of Guilt*	✓	✓	✓	✓
*3*	*Suicide*			✓	✓
*7*	*Work and activities*	✓	✓	✓	✓
*9*	*Agitation*		✓		✓
*10*	*Anxiety (Psychic)*	✓	✓	✓	✓
*11*	*Anxiety (Somatic)*	✓	✓	✓	✓
*13*	*Somatic Symptoms (General)*	✓		✓	
*14*	*Genital Symptoms*				✓
	Score range	0–22	0–22	0–26	0–28

MP-6 = Maier-Philip-6 subscale.

Item response analysis has demonstrated that HAMD items such as *Depressed Mood*, *Work and Activities*, *Guilt*, *Anxiety/Psychic*, *Anxiety/Somatic* and *Somatic/General* show good discriminative properties across almost the entire range of depression severity, and it is these items that most closely approximate the ‘‘ideal” item [9]. Subset scales containing these six items, e.g. the Evans-6 and Toronto-7, cover the important symptom domains of observed mood and interest-activity, which can predict treatment outcome with antidepressant drugs [14, 16–18]. Continued use of items insensitive to change may underestimate actual treatment effects, necessitating larger samples to demonstrate that an effect is statistically significant [7].

Various studies have examined the predictive value in ECT of depression severity rating scales such as the HAMD and Montgomery Asberg Depression Rating Scale (MADRS) [16, 19, 20]. To our knowledge, only one study to date has investigated the ability of a HAMD subset scale to predict outcome with ECT [16]. The study found the Toronto-7 to be a reliable and valid measure that was sensitive to changes in depressive symptoms following treatment with fluoxetine or ECT. They also reported that early improvement, as measured by either the HAMD-17 or Toronto-7, was capable of predicting response and remission to fluoxetine or ECT with good discriminative capacity. However, this secondary analysis was based on open label trial data. No controlled trial to date has compared the ability of HAMD subset scales to predict therapeutic outcomes with ECT.

For the present study, we used ECT patient data from the EFFECT-Dep trial [21] to investigate the most frequently used and validated subset scales (i.e. Maier-Phillip 6 item [12], Evans-6 [9], Toronto-7 [10] and Gibbons-8 [11]) for their predictive ability, as well as three “full” HAMD versions (HAMD-17, HAMD-21 [22] and HAMD-24 [23]). We did not include the Bech-6 as it does not incorporate the anxiety items (*Psychic* and *Somatic*) previously reported to predict treatment outcome [17, 18]. The Evans-6 was reported to slightly outperform the Bech-6 in terms of predictive capacity in recent analyses [14] and therefore was included instead.

We hypothesised that the Evans-6, Maier-Philip-6, Toronto-7 and Gibbons-8 subset scales at baseline would have good predictive ability for response, remission and percentage reduction in HAMD-24 scores from baseline to end of treatment (EOT) in an ECT population. We similarly examined full versions of the HAMD and explored the ability of individual items in predicting clinical outcomes with ECT.

## Materials and methods

### Participants

In-patients with depression (n = 138) were recruited for the EFFECT-DEP Trial (Enhancing the Effectiveness of ECT in Severe Depression; ISRCTN23577151; NCT01907217) [21]. Two people withdrew and therefore were excluded from the analysis. 136 participants (63% female; age = 56.7 years [SD = 14.8]) were entered into the analysis for this study. Briefly, the original trial was a pragmatic, patient and rater-blinded, two-group, parallel, randomized, noninferiority trial. The trial compared twice-weekly high-dose (6 x seizure threshold) right unilateral ECT (n = 67) with moderate-dose (1.5 x seizure threshold) bitemporal ECT (n = 69) in routine clinical practice and took place in St. Patrick’s Mental Health Services, Dublin, Ireland.

Eligible participants were aged ≥18 years, referred for ECT, met diagnostic criteria for a major depressive episode (unipolar or bipolar; Structured Clinical Interview for DSM-IV) [24] and scored ≥21 on the 24-item HAMD. Exclusion criteria were: conditions rendering patients unfit for general anaesthesia or ECT; ECT in previous 6 months; history of schizophrenia, schizoaffective disorder, or neurodegenerative or other neurological disorder; alcohol/substance abuse in previous 6 months; involuntary status; and inability/refusal to consent. Patients continued regular antidepressant treatments. This study was approved by the St. Patrick’s University Hospital Research Ethics Committee and written informed consent was obtained after procedures were fully explained.

### Electroconvulsive therapy

Using a stimulus dosing protocol, brief-pulse (1.0 msec pulse width; current amplitude 800mA) ECT was administered twice weekly (Mecta 5000M device, MectaCorp., Portland, Ore.; maximum 1200mC), using methohexital (0.75–1.0 mg/kg) anaesthesia and succinylcholine (0.5–1.0mg/kg) for muscle relaxation [21]. The number of ECT sessions was decided by referring clinicians and patients, up to a maximum of 12 sessions.

### Clinical assessments

Depression severity was measured by the HAMD-24 [23] and ratings were obtained at baseline, after every second ECT session (i.e. weekly), and soon after (2–4 days) completing the ECT course (end of treatment). Interrater reliability for HAM-D scoring was assessed every 6 months and the median intraclass correlation agreement was 0.96 (range: 0.89–0.98). The scale items for the 17, 21 and 24-item versions of the HAMD are shown in S1 Table. Response was defined as >60% decrease from baseline HAMD-24 score and a score <16, as is comparable with other relevant trials comparing bilateral and unilateral ECT2. Remission was defined as >60% decrease from baseline HAMD-24 score and a score <10 for two consecutive weeks. Scores for the four HAMD subset scales and the HAMD-17 and HAMD-21 were derived from the HAMD-24. Baseline Clinical Global Impression Severity (CGI-S) was rated by referring clinicians [23].

### Statistical analyses

Statistical analyses were performed with IBM SPSS Version 25 (IBM Corporation, NY, USA). Variables were examined for normality using measures of skewness and kurtosis, Q-Q plots, box plots and histograms. Analyses were conducted on the intention to treat principle and the threshold for statistical significance was set at <0.05. Benjamini Hochberg’s correction was used for analyses where a pre-determined hypothesis was stated. In this case, that the Evans-6, Maier-Philip-6, Toronto-7 and Gibbons-8 subset scales at baseline would have good predictive ability for response, remission and percentage reduction in HAMD-24 scores from baseline to end of treatment (EOT) in an ECT population. We similarly examined full versions of the HAMD for predictive ability for response and remission [25]. No correction for multiple comparisons was made for the exploratory analyses [26].

Independent sample t-tests were performed to determine any differences in baseline depression severity scores between responders and non-responders as well as remitters and non-remitters. Associations between HAMD subset scales and full versions were evaluated using Pearson product moment correlations. A Pearson correlation coefficient (r) value of > 0.70 indicates a strong association [27]. Understanding the scales’ relationships with one another is useful for using the value of one scale to predict the value of another.

Concurrent validity was assessed using two-tailed Pearson’s product moment correlations to determine the relationship between HAMD subset scales and full versions with the Clinical Global Impression severity scale (CGI-S) at baseline. Reliability, a measure of internal consistency, was assessed using a measure of Cronbach’s alpha.

Simple logistic regression models were fitted to the data with baseline HAMD subset scale scores as predictor variables and response status (yes/no dichotomous variable) as the outcome variable with laterality added as a covariate to control for any effects due to different electrode placements. The same was performed for remitter status. Receiver operating characteristics (ROC) analyses were conducted to examine the specificity and sensitivity of the subset scales for predicting response and remission. To investigate the ability of HAMD subset scales at baseline to predict percentage reduction in depression severity, as measured by the HAMD-24, simple linear regression models were fitted with the various subset scales as predictors and percentage reduction in HAMD-24 scores from baseline to end of treatment (EOT) as the outcome variable.

To evaluate the ability of early improvement to predict response and remission after acute treatment, we calculated the sensitivity, specificity, positive predictive value (PPV) and negative predictive value (NPV) of early improvement as measured by a >20% decrease in the HAMD subset scales and the three full versions after two weeks of ECT (i.e. four sessions). This is considered a standard measure of early improvement for subset scales and full versions of the HAMD [14].

For exploratory analyses, each item of the HAMD-24 at baseline was entered into an individual logistic regression model to investigate which items were significantly associated with response and remission.

## Results

### Normality tests

The Kolmogrov-Smirnov test for the four subset scales and three full versions at baseline was found to be significant, suggesting that the assumption of normality was violated in the sample. However, this test is affected by sample size and thus requires assessing normality in other ways. The measures of skewness and kurtosis, box plot and Q-Q plots were all found to be within normal distribution parameters. Therefore, the data were treated as being normally distributed.

### Baseline depression severity

Baseline HAMD scores of all responder and remitter groups are shown in Table 2. Independent samples t-tests revealed no statistically significant differences between responders and non-responders or remitters and non-remitters at baseline on any of the subset scales or full versions of the HAMD.

**Table 2:** Baseline depression severity for responders and remitters.

**Subscale**	**N**	**Responders** Mean (SD)	**N**	**Non-responders** Mean (SD)	**t**	**df**	***p***
*Evans-6*	76	13.22 (2.56)	60	12.98 (2.26)	0.57	134	0.57
*MP-6*	76	12.72 (2.87)	60	11.93 (2.44)	1.70	134	0.09
*Toronto-7*	76	14.63 (3.11)	60	14.83 (2.73)	-0.40	134	0.69
*Gibbons-8*	76	15.14 (3.34)	60	14.00 (3.02)	0.20	134	0.84
*HAMD-17*	76	22.01 (4.92)	60	21.02 (3.83)	1.29	134	0.19
*HAMD-21*	76	24.51 (5.44)	60	23.68 (4.39)	0.95	134	0.35
*HAMD-24*	76	30.43 (6.61)	60	29.43 (5.65)	0.93	134	0.35
**Subscale**	**N**	**Remitters** Mean (SD)	**N**	**Non-remitters** Mean (SD)	**t**	**df**	***p***
*Evans-6*	61	12.98 (2.53)	75	13.22 (2.36)	-0.58	134	0.56
*MP-6*	61	12.55 (2.74)	75	12.22 (2.70)	0.71	134	0.48
*Toronto-7*	61	14.32 (3.08)	75	15.04 (2.80)	-1.41	134	0.16
*Gibbons-8*	61	14.84 (3.41)	75	15.31 (3.26)	-0.85	134	0.37
*HAMD-17*	61	21.39 (4.87)	75	21.72 (4.12)	-0.42	134	0.67
*HAMD-21*	61	23.85 (5.31)	75	24.38 (4.79)	-0.61	134	0.54
*HAMD-24*	61	29.64 (6.11)	75	30.28 (6.29)	-0.60	134	0.55

### Relationships between the HAMD subset scales and full versions

A one-tailed Pearson’s product moment correlation determined that all subset scales and full versions have moderate to strong (all ≥ 0.62) positive relationships with each other (*p* ≤0.05), meaning the subset scales were all highly related with one another, suggesting they measure the same construct (S2 Table).

### Reliability and concurrent validity of HAMD subset scales and full versions

Reliability analyses revealed that at baseline, the subset scales and full versions were unsatisfactory, with Cronbach’s alpha values between 0.22 and 0.53 for all subset scales and full versions (Table 3). At baseline, five of the seven subset scales and full versions demonstrated weak, positive correlations with the CGI-S (Table 3). The HAMD-17 and HAMD-21 were not significantly correlated with the CGI-S. This suggests poor concurrent validity of these versions with an alternative illness severity rating scale, the CGI-S.

**Table 3:** Reliability and concurrent validity measures of the HAMD subscales and full versions at baseline.

	Evans-6	MP-6	Toronto-7	Gibbons-8	HAMD-17	HAMD-21	HAMD-24
Cronbach’s alpha	0.22	0.37	0.34	0.33	0.38	0.42	0.53
r^a^	0.23**	0.24**	0.23**	0.23**	0.15	0.15	0.18*

MP-6 = Maier-Philip-6 subscale

^a^correlation with the CGI severity scale

*p ≤ 0.05

**p ≤ 0.01.

### Predictors of response, remission and percentage reduction in HAMD-24

Results of simple logistic regression analyses performed on the data with response and remission as the outcome variables are presented in Table 4. None of the four HAMD subset scales or full versions at baseline were significantly associated with the odds of being a responder or a remitter. In line with the original trial’s definition of response, we also used a 60% decline from baseline to EOT score criterion to determine responder status for each of the subset scales. We found no significant association between the subset scales at baseline and any subset scale-based responder status (see S3 Table). We also investigated whether the CGI-S at baseline was associated with response or remission and found there to be no association (see S4 Table).

**Table 4:** Baseline HAMD subscales and full versions as predictors of response and remission.

Subscale	β	OR	95% CI	*p*
**Response**				
*Evans-6*	-0.38	0.96	0.84–1.11	0.60
*MP-6*	-0.11	0.89	0.78–1.02	0.09
*Toronto-7*	0.02	1.02	0.91–1.15	0.70
*Gibbons-8*	-0.02	0.98	0.89–1.09	0.78
*HAMD-17*	-0.05	0.95	0.88–1.03	0.22
*HAMD-21*	-0.03	0.97	0.91–1.04	0.39
*HAMD-24*	-0.02	0.97	0.92–1.03	0.40
**Remission**				
*Evans-6*	0.04	1.05	0.91–1.20	0.54
*MP-6*	-0.05	0.96	0.84–1.08	0.48
*Toronto-7*	0.08	1.09	0.96–1.22	0.16
*Gibbons-8*	0.05	1.05	0.94–1.16	0.41
*HAMD-17*	0.02	1.02	0.94–1.09	0.64
*HAMD-21*	0.02	1.02	0.96–1.09	0.50
*HAMD-24*	0.02	1.02	0.96–1.08	0.51

MP-6 = Maier-Philip-6 subscale, OR = Odds ratio.

Results of the linear regression analyses performed on the data with the percentage of HAMD-24 decrease from baseline to end of treatment as the outcome variable are presented in S5 Table. After corrections for multiple testing, only the Maier-Philip-6 remained significantly associated with percentage reduction in HAMD-24 scores from baseline to EOT. Higher baseline scores on the Maier-Philips-6 were associated with a greater percentage reduction in HAMD-24 scores from baseline to EOT. All other subset scales and full versions were not significantly associated with percentage reduction in HAMD-24 scores.

The four subset scales and three full versions of the HAMD were entered into ROC analyses to determine which had better discriminative capacity (i.e., which was better at predicting response and remission). An area under the curve (AUC) value of 0.7–0.8 indicates acceptable discrimination [28]. None of the AUC values for the subset scales or full versions demonstrated adequate specificity or sensitivity for correctly predicting response or remission in this sample of ECT patients (S6 Table).

### Predictive values for early improvement with ECT

Table 5 details the sensitivity, specificity, positive predictive values (PPV) and negative predictive values (NPV) for early improvement as assessed by the HAMD subset scales and full versions as a predictor of response and remission in patients receiving ECT. Sensitivity and specificity of ≥0.70 are considered high. The prevalence of achieving response at EOT within early improvers was between 52.1% and 56.3% for all the subset scales. The prevalence of achieving remission at EOT within early improvers was between 40.3% and 45.2% for all of the subset scales. All of the subset scales and full versions showed moderate to high (0.50–0.98) sensitivities, indicating that early improvement on these subset scales and full versions are moderately to highly sensitive predictors of response and remission in patients receiving ECT. With regards to specificity, the subset scales and full versions ranged from low to high specificity (0.06–0.83). The Evans-6, Toronto-7 and the HAMD-17 had the highest specificities, suggesting that early improvement on these subset scales and full version are moderately to highly specific predictors of response and remission in patients receiving ECT.

**Table 5:** Sensitivity, specificity, positive predictive values (PPV) and negative predictive values (NPV) for early improvement on the HAMD subscales and full versions to predict response and remission.

Measure	Outcome	PPV	NPV	Sensitivity	Specificity
Evans-6	Response	0.69	0.61	0.64	0.66
	Remission	0.57	0.71	0.67	0.62
MP-6	Response	0.56	0.44	0.80	0.20
	Remission	0.45	0.56	0.80	0.20
Toronto-7	Response	0.71	0.69	0.75	0.64
	Remission	0.61	0.80	0.80	0.61
Gibbons-8	Response	0.55	0.33	0.89	0.06
	Remission	0.44	0.42	0.88	0.07
HAMD-17	Response	0.77	0.58	0.50	0.83
	Remission	0.64	0.68	0.52	0.78
HAMD-21	Response	0.61	0.69	0.82	0.42
	Remission	0.49	0.80	0.85	0.39
HAMD-24	Response	0.61	0.83	0.94	0.32
	Remission	0.51	0.96	0.98	0.30

MP-6 = Maier-Philip-6 subscale.

A high PPV is considered to be 0.7 or above [16]. All versions ranged from low to high PPVs (0.44–0.71), with the Gibbons-8, the Maier-Philip-6 and the HAMD-21 having the lowest PPVs for response and remission (see Table 5). This suggests that, with the exception of these three versions, early improvement as defined by the subset scales and full versions of the HAMD is a moderately to highly positive predictor of achieving response and remission. With regards to NPV, the values ranged from low to high (0.33–0.96) for all versions, with the Maier-Philip-6 and Gibbons-8 having the lowest values. These results suggest that with the exception of these two subset scales, patients who did not exhibit early improvement as defined by the subset scales and full versions of the HAMD had a moderate to low likelihood of achieving response and remission.

### Exploratory analyses

Looking to the individual items within the HAMD-24 and associated subset scales, simple logistic regression analyses revealed that at baseline four of the 24 items were significantly associated with response: *Agitation*, *Somatic Symptoms (General)*, *Hypochondriasis* and *Suicide*. With regards to remission, only *Somatic Symptoms (General)* was found to be significantly associated with remission (Table 6).

**Table 6:** Individual HAMD-24 items as predictors of response.

Item	β	OR	95% CI	*p*	β	OR	95% CI	*p*
**RESPONSE**					**REMISSION**			
*1*. *Depressed Mood*	-0.38	0.70	0.44–1.06	0.92	-0.38	0.68	0.40–1.17	0.16
*2*. *Feelings of Guilt*	0.02	1.02	0.70–1.48	0.91	0.23	1.25	0.76–2.06	0.38
*3*. *Suicide*	0.33	1.40	1.02–1.91	0.04*	0.23	1.26	0.83–1.91	0.29
*4*. *Insomnia (early)*	-0.22	0.82	0.55–1.21	0.31	0.04	1.05	0.64–1.72	0.86
*5*. *Insomnia (middle)*	0.04	1.04	0.69–1.56	0.86	-0.03	0.98	0.56–1.70	0.93
*6*. *Insomnia (Late)*	0.14	1.15	0.74–1.79	0.53	0.29	1.35	0.74–2.47	0.33
*7*. *Work and Activities*	-0.10	0.90	0.58–1.40	0.65	-0.07	0.93	0.53–1.65	0.82
*8*. *Psychomotor Retardation*	-0.36	0.70	0.45–1.08	0.11	-0.06	0.95	0.55–1.62	0.84
*9*. *Agitation*	-0.54	0.58	0.37–0.93	0.02*	-0.19	0.82	0.49–1.40	0.47
*10*. *Anxiety (Psychic)*	-0.21	0.81	0.62–1.05	0.12	0.06	1.06	0.74–1.50	0.76
*11*. *Anxiety (Somatic)*	0.32	1.34	0.86–2.19	0.18	0.28	1.33	0.76–2.31	0.32
*12*. *Somatic Symptoms (GI)*	-0.17	0.85	0.54–1.32	0.46	-0.06	0.95	0.54–1.66	0.84
*13*. *Somatic Symptoms (General)*	0.65	1.92	1.01–3.5	0.03*	0.71	2.03	0.99–4.15	0.05*
*14*. *Genital Symptoms*	0.26	1.30	0.90–1.87	0.16	0.38	1.47	0.94–2.30	0.10
*15*. *Hypochondriasis*	-0.32	0.72	0.52–0.99	0.05*	-0.28	0.76	0.51–1.12	0.16
*16*. *Insight*	-20.39	0.00	0.00	0.99	-0.90	0.41	0.11–1.49	0.17
*17*. *Weight loss*	-0.22	0.80	0.47–1.36	0.27	-0.00	0.99	0.50–1.98	1.00
*18(a)*. *Diurnal variation (Time)*	0.22	1.24	0.75–2.01	0.41	-0.26	0.77	0.31–1.94	0.58
*18(b)*. *Diurnal variation (Severity)*	0.13	1.13	0.76–1.70	0.54	0.10	1.11	0.53–2.29	0.79
*19*. *Depersonalisation or derealisation*	0.15	1.17	0.74–1.84	0.51	-0.01	0.99	0.55–1.80	0.98
*20*. *Paranoid Symptoms*	-0.26	0.77	0.46–1.27	0.31	-0.23	0.80	0.45–1.43	0.44
*21*. *Compulsive Symptoms*	0.22	1.20	0.67–2.31	0.49	0.40	1.49	0.64–3.45	0.36
*22*. *Helplessness*	-0.06	0.94	0.69–1.27	0.69	0.13	1.14	0.75–1.73	0.54
*23*. *Hopelessness*	0.06	1.06	0.76–1.49	0.79	0.08	1.08	0.69–1.68	0.74
*24*. *Worthlessness*	0.09	1.10	0.82–1.49	0.53	-0.13	0.87	0.69–1.68	0.53

## Discussion

To our knowledge, this is the first report to compare the predictive ability of multiple HAMD subset scales and various full versions in a depressed population treated with ECT. Contrary to our hypothesis, the predictive performance from baseline to EOT was poor for all subset scales and full versions, with AUCs between 51% and 57% for response and 47% and 54% for remission. Additionally, concurrent validity findings were not considered acceptable. The subset scales demonstrated weak correlations with the CGI-S. A high correlation is often regarded as evidence that two rating scales measure the same clinical factor. Low correlations between the two may reflect inherent fallibility in the HAMD full versions and subset scales as concurrent validity findings have been mixed [29].

Our reliability findings are lower than the accepted value for Cronbach’s alpha; in fact, they were unsatisfactory. However, this compares similarly to other studies using these subset scales. When these are used in either ECT [16] or antidepressant populations [20], the Cronbach’s alpha varies from 0.44 to 0.71, thus suggesting that they were mostly unreliable in ECT and also in antidepressant study populations. Indeed, it would appear that previous assessments of the subset scales have found them to be unsatisfactory and our findings suggest this is even more so in an ECT population.

Our findings are in agreement with that of a previous review of depression rating scales, including the HAMD [30]. This review commented on the limitations of the common assumption that depression sum-scores adequately represent the severity of one underlying condition. It seems unlikely that depression symptoms are interchangeable measurements of one depression construct due to their pronounced differences in relation to important constructs. Our findings of low reliability reflect this inadequacy of sum-total scores to accurately describe the severity of disease, i.e. depression. Depression scores would be better thought of as composite scores of psychopathological problems due to a condition called ‘depression’ [31]. It is difficult to quantitatively measure someone’s depression, so we use these rating scales to do so. However, when we do, we ignore the psychometric shortcomings that these measures clearly have.

A recent meta-analysis found depression severity at baseline to be a predictor of response in ECT though with only a modest effect size (SMD = 0.19) [3]. Other studies have found no predictive power for response with respect to depression severity in an ECT population [32, 33]. Depression severity based on HAMD scales and, as shown here, the CGI-S may therefore not be a robust predictor of response and remission in ECT.

All of the HAMD subset scales and full versions at baseline, with the exception of the Maier-Philip-6, were found not to be significantly associated with percentage reduction in HAMD-24 score from baseline to EOT. This suggests that the Maier-Philip-6 subset scale could have some clinical utility for predicting therapeutic outcome, even though it was not found to be predictive of response or remission. However, use of dichotomous outcome measures such as response and remission have been reported as better reflective of clinical reality than average HAMD differences, because the latter metric implies that all treated patients will experience the same treatment effect [34].

Other studies have found the subset scales’ predictive power to be as effective at predicting response and remission in patients with a major depressive episode as the HAMD-17 [35]. However, most of these studies used antidepressant clinical trial populations as opposed to an ECT population [6, 15, 35]. Comparative results found that certain subset scales are better able to detect antidepressant treatment effects than the full scale depending on the type of treatment (drugs, psychotherapy, etc.) and pharmacological mechanism of action of the drug administered [15, 18]. This could suggest that the HAMD subset scales at baseline have adequate predictive ability in an antidepressant drug population but lack this for ECT. One possibility is that patients enrolled in antidepressant trials are different to those enrolled in ECT trials. Further dedicated analysis would be required to confirm this.

Regarding early improvement as detected by the HAMD subset scales, we found the Evans-6, Toronto-7, HAMD-17 and HAMD-21 were sensitive and specific predictors of response and remission, suggesting some clinical utility. Lin et al (2019) investigated the predictive ability of the Toronto-7 in an ECT population had similar findings [16]. They found early improvement, as measured by either the HAMD-17 or Toronto-7, was capable of predicting response and remission to acute treatment with fluoxetine or ECT with good discriminative capacity. Our findings are consistent with that of Lin et al with regards early improvement, with the exception of the HAMD-17, which was found to have poor sensitivity in our study. Four of the seven subset scales and full versions had moderate (0.4–0.69) to high (≥0.7) PPVs and NPVs, suggesting that early improvement is associated with increased likelihood of achieving response and remission. As aforementioned, these results are consistent with the results of several other antidepressant studies showing that early improvement was associated with increased likelihood of achieving response and/or remission in patients with depression [16, 36].

In terms of the exploratory element of this study, several items of the HAMD-24 at baseline were found to have adequate predictive ability. The *Suicide*, *Agitation*, *Hypochondriasis* and *Somatic Symptoms [General]* items were all significantly associated with response. *Somatic Symptoms [General]* also demonstrated predictive ability for achieving remission. As specific items have different predictive abilities [9], these results may be indicative of items that have better predictive ability in an ECT population. Of note, only two of the four subset scales (Evans-6 and Toronto-7) include *Somatic Symptoms (General*), only two (Gibbons-8 and Toronto-7) include *Suicide*, only one (Gibbons-8) includes *Agitation* and none include *Hypochondriasis*. A common criticism of the HAMD is that some of the items measure single symptoms along a meaningful continuum of severity but that many do not. The *Somatic Symptoms (General)* item, which is also symptomatically heterogeneous, includes feelings of heaviness, diffuse backache, and loss of energy.^7^ The intrinsic problems in the heterogeneity of these rating descriptors detract from the potential meaningfulness of these items, a problem worsened if the different components of an item actually measure multiple constructs and thus measure different effects. This could mitigate the suspected predictive ability of the *Somatic Symptoms* item as found in our exploratory analysis. Previous analyses have also found the use of items such as *Somatic Symptoms* to be problematic in older populations, as they may be capturing age-related physical illness effects as opposed to depressive symptoms [37]. As these were exploratory analyses and we did not correct for multiple comparisons, these findings should be interpreted with caution.

Psychomotor disturbance as measured by the 18-item CORE assessment has been reported to be a predictor of response (OR = 0.84) in an ECT population (N = 77) [38]. In contrast, in our population (N = 136) we found agitation as measured by the *Agitation* item of the HAMD-24 scale to be associated with response but not remission. The *Psychomotor Retardation* item was not found to be significantly associated with either outcome. Again, we interpret this finding as a reflection of the HAMD shortcomings in accurately measuring depressive symptoms as other more dedicated measures such as the CORE assessment can.

### Limitations

A limitation of our study was that all participants in the trial were in-patients and this may not therefore be generalisable to ECT practice elsewhere. However, because of its pragmatic design, the trial does reflect real world practice and had excellent adherence and retention [21]. This was a secondary analysis of trial data and therefore the original trial was not specifically designed for the analyses conducted here. A third limitation stems from the non-independence of the subset scales. All four versions are nested within the HAMD-17 and do not contain any of the additional items found within the HAMD-21 or HAMD-24, although none of these additional items proved to have any individual predictive ability. Most research investigating therapeutic outcome prediction has used the HAMD-17 as the primary outcome and for defining response and remission [3, 16, 20]. Fourthly, there was reduced variability in HAMD-24 scores at baseline as an inclusion criterion for entry to the original EFFECT-Dep trial was a score of ≥21 on the HAMD-24, meaning there was a limited lower range. Of note, such patients accounted for only 29 (13.1%) of the 222 patients who were excluded from participation in the trial [21]. Thus, the inferences made in this study may not apply to those ECT patients with depression who score lower than 21 on the HAMD-24.

## Conclusion

The lack of reliability and predictive validity of any of the HAMD subset scales or full versions at baseline for response and remission highlights an inherent weakness of the HAMD in an ECT setting. However, the subset scales showed some clinical utility regarding early improvement and percentage reduction in HAMD-24 scores from baseline to end of ECT course that might be useful for guiding treatment. We support the need for more tailored depression rating scales for ECT.

## Supporting information

S1 Table Items of the HAMD-17, HAMD-21 and HAMD-24. (DOCX)

S2 Table Correlations between HAMD subscales and full versions. (DOCX)

S3 Table Subset scales as predictors of response with subset scale-based responder status as outcome. (DOCX)

S4 Table Baseline CGI-S as a predictor of response and remission. (DOCX)

S5 Table Baseline HAMD subscale and full version scores as predictors of percentage reduction of HAMD-24 from baseline to EOT. (DOCX)

S6 Table Results of the ROC analysis for HAMD subscales and full versions at baseline. (DOCX)

S1 Dataset (SAV)
