# Supplementary material for: Usefulness of Hamilton rating scale for depression subset scales and full versions for electroconvulsive therapy
Source: PLoS One. 2021 Nov 9;16(11):e0259861. doi: 10.1371/journal.pone.0259861 (PMC8577745; doi:10.1371/journal.pone.0259861)
Supplement: S1 Table — (DOCX) [file pone.0259861.s001.docx]

**TABLE S1**: *Items of the HAMD-17, HAMD-21 and HAMD-24*

|  | **HAMD-17** | **HAMD-21** | **HAMD-24** |
| --- | --- | --- | --- |
| HAMD-24 Item No. |  | Items |  |
| 1 | *Depressed mood* | *Depressed Mood* | *Depressed Mood* |
| 2 | *Feelings of guilt* | *Feelings of guilt* | *Feelings of guilt* |
| 3 | *Suicide* | *Suicide* | *Suicide* |
| 4 | *Insomnia (initial)* | *Insomnia (initial)* | *Insomnia (initial)* |
| 5 | *Insomnia (middle)* | *Insomnia (middle)* | *Insomnia (middle)* |
| 6 | *Insomnia (late)* | *Insomnia (late)* | *Insomnia (late)* |
| 7 | *Work and activities* | *Work and activities* | *Work and activities* |
| 8 | *Retardation* | *Retardation* | *Retardation* |
| 9 | *Agitation* | *Agitation* | *Agitation* |
| 10 | *Anxiety (psychic)* | *Anxiety (psychic)* | *Anxiety (psychic)* |
| 11 | *Anxiety (somatic)* | *Anxiety (somatic)* | *Anxiety (somatic)* |
| 12 | *Somatic symptoms (GI)* | *Somatic symptoms (GI)* | *Somatic symptoms (GI)* |
| 13 | *Somatic symptoms (General)* | *Somatic symptoms (General)* | *Somatic symptoms (General)* |
| 14 | *Genital symptoms* | *Genital symptoms* | *Genital symptoms* |
| 15 | *Hypochondriasis* | *Hypochondriasis* | *Hypochondriasis* |
| 16 | *Insight* | *Insight* | *Insight* |
| 17 | *Weight loss* | *Weight loss* | *Weight loss* |
| 18 |  | *Diurnal variation* | *Diurnal variation* |
| 19 |  | *Depersonalisation and derealisation* | *Depersonalisation and derealisation* |
| 20 |  | *Paranoid symptoms* | *Paranoid symptoms* |
| 21 |  | *Obsessional symptoms* | *Obsessional symptoms* |
| 22 |  |  | *Helplessness* |
| 23 |  |  | *Hopelessness* |
| 24 |  |  | *Worthlessness* |
| **Score range** | 0-54 | 0-64 | 0-76 |
