# Supplementary material for: Usefulness of Hamilton rating scale for depression subset scales and full versions for electroconvulsive therapy
Source: PLoS One. 2021 Nov 9;16(11):e0259861. doi: 10.1371/journal.pone.0259861 (PMC8577745; doi:10.1371/journal.pone.0259861)
Supplement: S2 Table — (DOCX) [file pone.0259861.s002.docx]

**TABLE S2:** *Correlations between HAMD subscales and full versions*

|  | *Evans-6* | *MP-6* | *Toronto-7* | *Gibbons-8* | *HAMD-17* | *HAMD-21* | *HAMD-24* |
| --- | --- | --- | --- | --- | --- | --- | --- |
| *Evans-6* | 1 | 0.91** | 0.93** | 0.88** | 0.70** | 0.69** | 0.67** |
| *MP-6* | 0.91** | 1 | 0.82** | 0.92** | 0.68** | 0.64** | 0.62** |
| *Toronto-7* | 0.93** | 0.82** | 1 | 0.92** | 0.74** | 0.73** | 0.88** |
| *Gibbons-8* | 0.88** | 0.92** | 0.92** | 1 | 0.77** | 0.73** | 0.73** |
| *HAMD-17* | 0.70** | 0.68** | 0.74** | 0.77* | 1 | 0.93** | 0.88** |
| *HAMD-21* | 0.69** | 0.64** | 0.73** | 0.73** | 0.93** | 1 | 0.92** |
| *HAMD-24* | 0.67** | 0.62** | 0.88** | 0.73** | 0.88** | 0.92** | 1 |
| MP-6 = Maier-Philip-6 subscale. **p ≤ 0.001. * ≤ 0.05 | | | | | | | |
