# Supplementary material for: Usefulness of Hamilton rating scale for depression subset scales and full versions for electroconvulsive therapy
Source: PLoS One. 2021 Nov 9;16(11):e0259861. doi: 10.1371/journal.pone.0259861 (PMC8577745; doi:10.1371/journal.pone.0259861)
Supplement: S3 Table — (DOCX) [file pone.0259861.s003.docx]

| **Table S3**: *Subset Scales as Predictors of Response with Subset Scale-based Responder Status as Outcome* | | | | |
| --- | --- | --- | --- | --- |
| **Subscale** | **β** | **OR** | **95% CI** | ***p*** |
| **Response (≥60% decline from baseline score)** | | | | |
| *Evans-6* | 0.009 | 1.01 | 0.88-1.16 | 0.84 |
| *MP-6* | -0.09 | 0.91 | 0.80-1.03 | 0.14 |
| *Toronto-7* | -0.02 | 0.98 | 0.87-1.10 | 0.75 |
| *Gibbons-8* | -0.09 | 0.91 | 0.82-1.01 | 0.84 |

*Statistical analysis: Logistic regression with subset scales as predictors and subset-scale based responder status*
