# Supplementary material for: Usefulness of Hamilton rating scale for depression subset scales and full versions for electroconvulsive therapy
Source: PLoS One. 2021 Nov 9;16(11):e0259861. doi: 10.1371/journal.pone.0259861 (PMC8577745; doi:10.1371/journal.pone.0259861)
Supplement: S4 Table — (DOCX) [file pone.0259861.s004.docx]

**Table S4**: *Baseline CGI-S as a predictor of response and remission*

|  | **β** | **OR** | **95% CI** | ***p*** |
| --- | --- | --- | --- | --- |
| Response | -0.40 | 0.67 | 0.41 – 1.08 | 0.11 |
| Remission | -0.34 | 0.71 | 0.44 – 1.14 | 0.16 |

*Statistical analysis: Logistic regression with CGI-S as predictor and response and remission status as outcome*
