# Supplementary material for: Usefulness of Hamilton rating scale for depression subset scales and full versions for electroconvulsive therapy
Source: PLoS One. 2021 Nov 9;16(11):e0259861. doi: 10.1371/journal.pone.0259861 (PMC8577745; doi:10.1371/journal.pone.0259861)
Supplement: S5 Table — (DOCX) [file pone.0259861.s005.docx]

**TABLE S5**: *Baseline HAMD subscale and full version scores as predictors of percentage reduction of HAMD-24 from baseline to EOT*

| ***Predictors*** | ***F-ratio*** | ***p*** | $\boldsymbol{R}^{\boldsymbol{2}}$ |
| --- | --- | --- | --- |
| ***Evans-6*** | 3.70 | 0.21 | 0.03 |
| ***MP-6*** | 8.10 | 0.03* | 0.06 |
| ***Toronto-7*** | 3.66 | 0.25 | 0.03 |
| ***Gibbons-8*** | 6.65 | 0.07 | 0.05 |
| ***HAMD-17*** | 3.32 | 0.14 | 0.05 |
| ***HAMD-21*** | 6.98 | 0.12 | 0.05 |
| ***HAMD-24*** | 8.68 | 0.17 | 0.06 |
| MP-6 = Maier-Philip-6 subscale, * = p ≤ 0.05 | | | |

*Statistical analysis: Logistic regression with subset scales and full versions as predictors and percentage reduction in HAMD scores as outcome*
