# Supplementary material for: Usefulness of Hamilton rating scale for depression subset scales and full versions for electroconvulsive therapy
Source: PLoS One. 2021 Nov 9;16(11):e0259861. doi: 10.1371/journal.pone.0259861 (PMC8577745; doi:10.1371/journal.pone.0259861)
Supplement: S6 Table — (DOCX) [file pone.0259861.s006.docx]

**TABLE S6:** *Results of the ROC analysis for HAMD subscales and full versions at baseline*

|  | **Response** | **Remission** |
| --- | --- | --- |
|  | AUC | AUC |
| *Evans-6* | 0.54 | 0.48 |
| *MP-6* | 0.57 | 0.54 |
| *Toronto-7* | 0.50 | 0.45 |
| *Gibbons-8* | 0.51 | 0.46 |
| *HAMD-17* | 0.55 | 0.46 |
| *HAMD-21* | 0.54 | 0.46 |
| *HAMD-24* | 0.53 | 0.47 |
| MP-6 = Maier-Philip-6 subscale | | |
